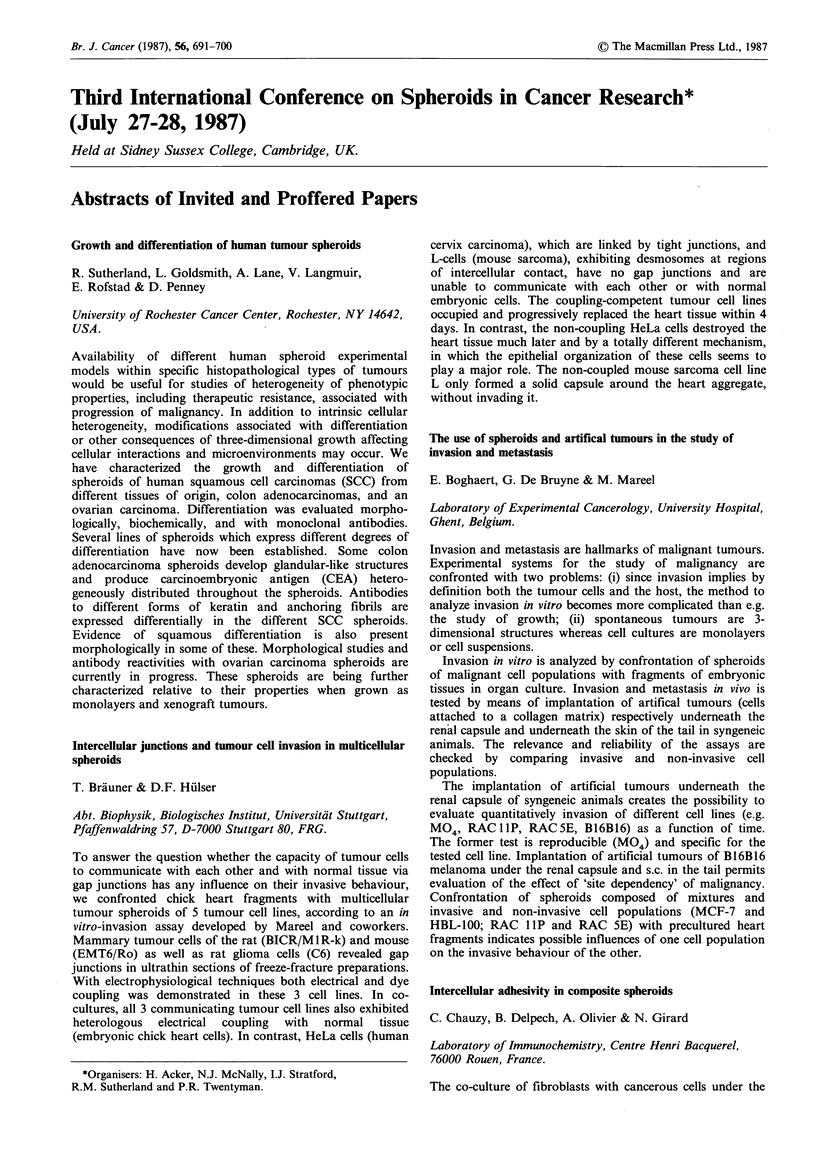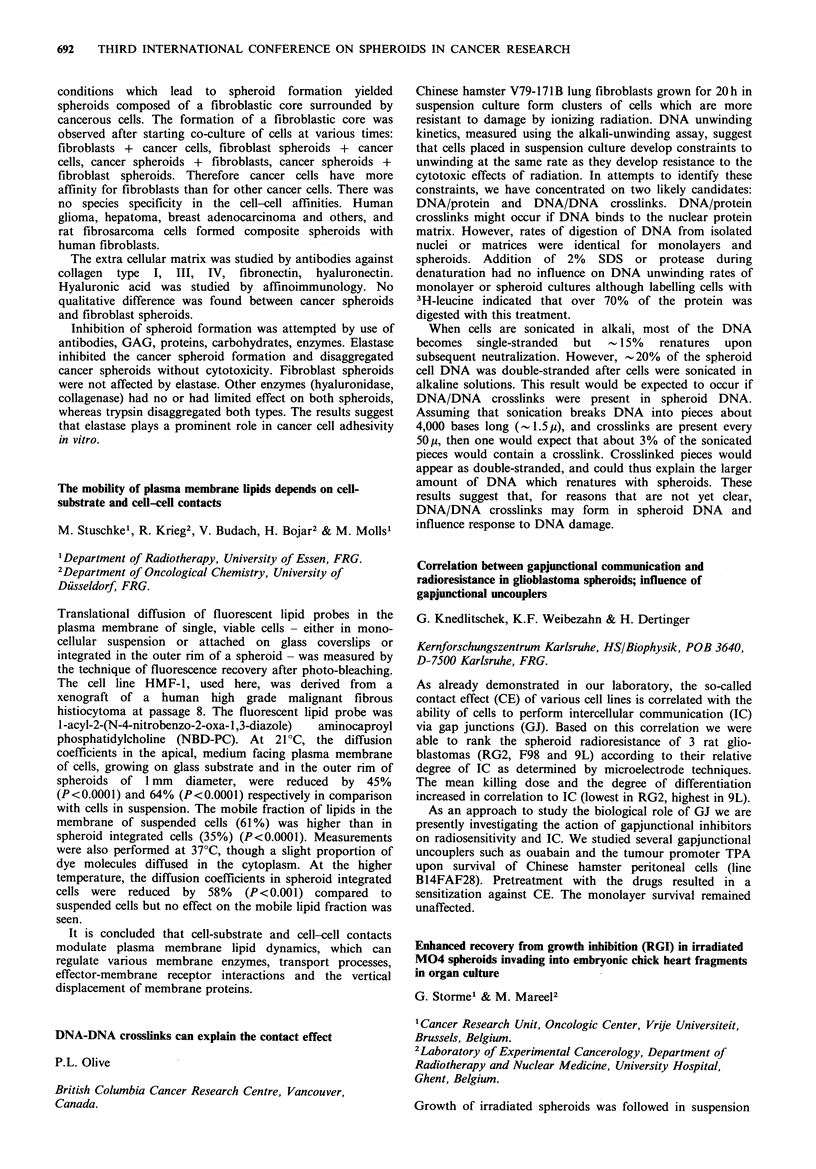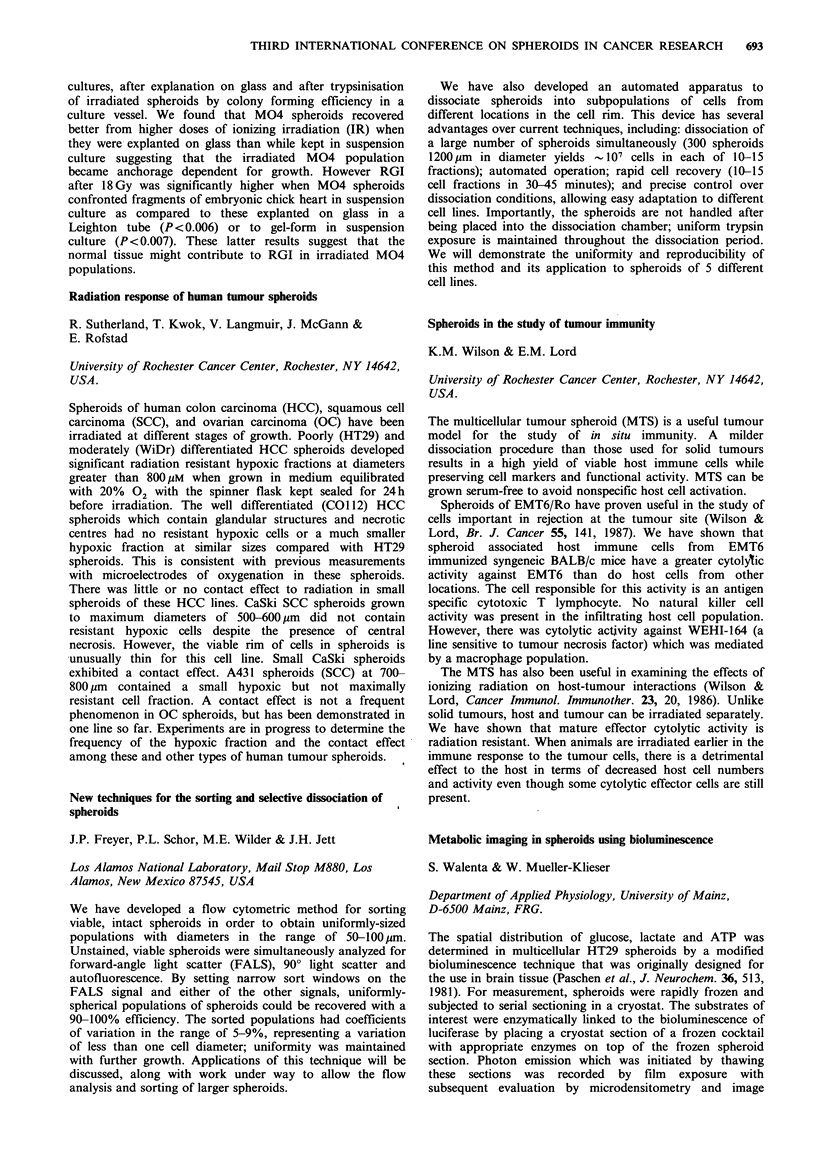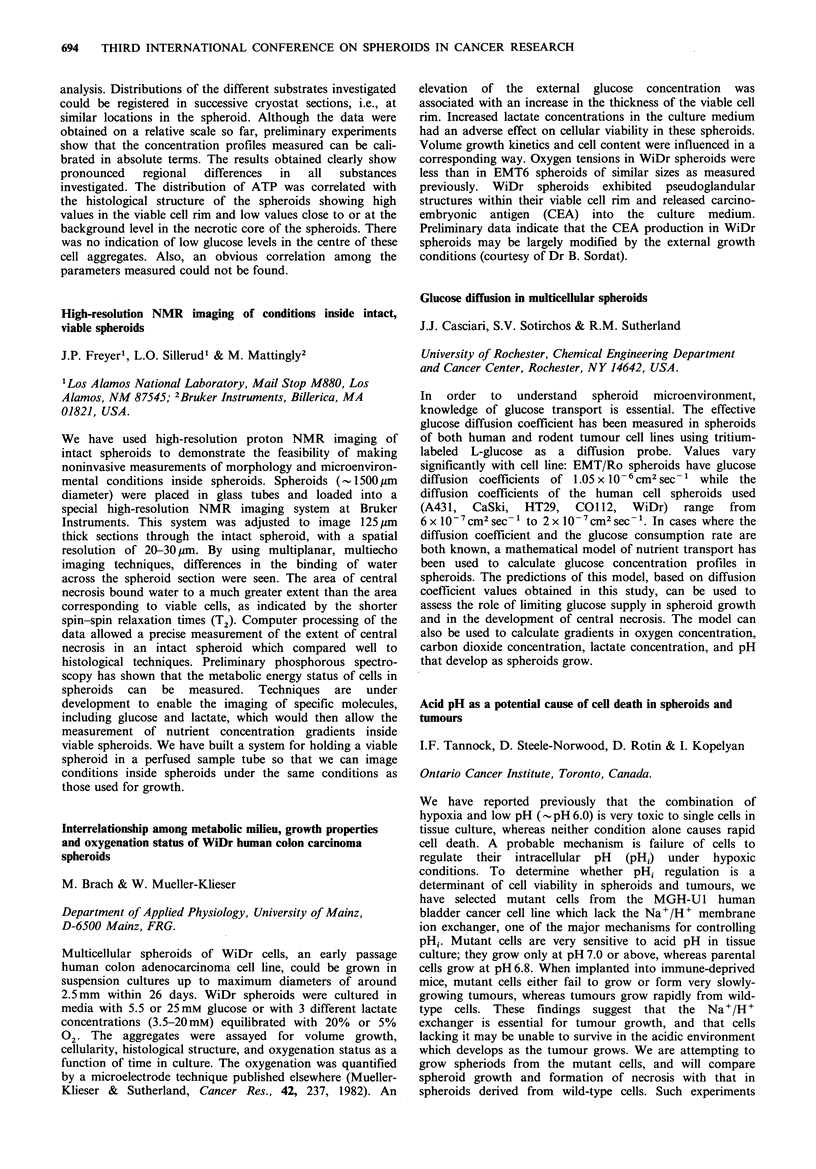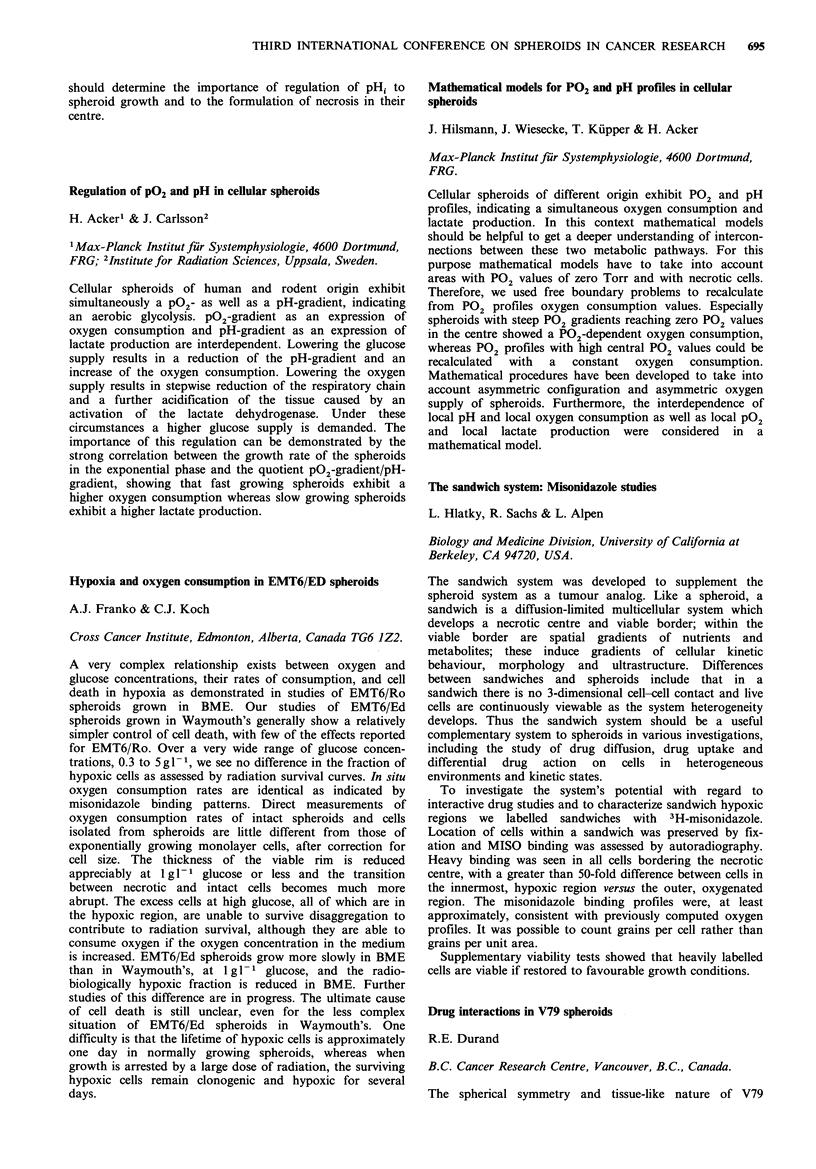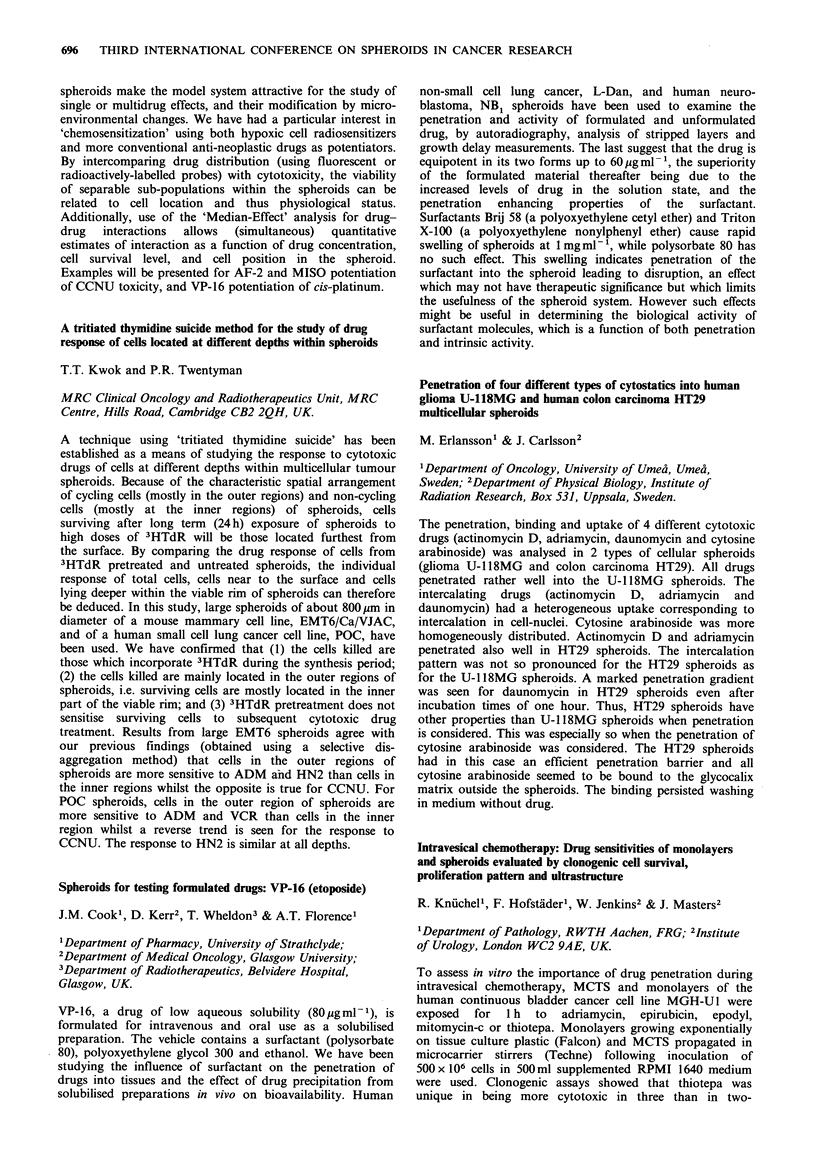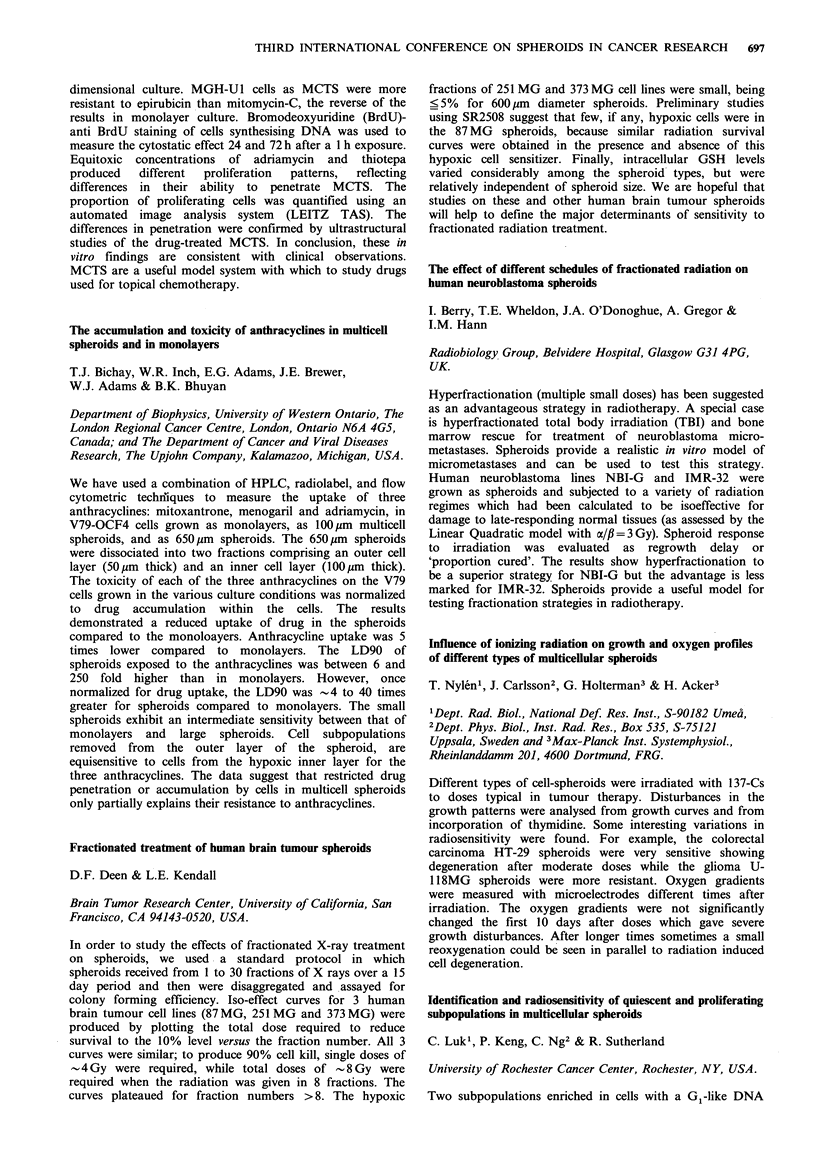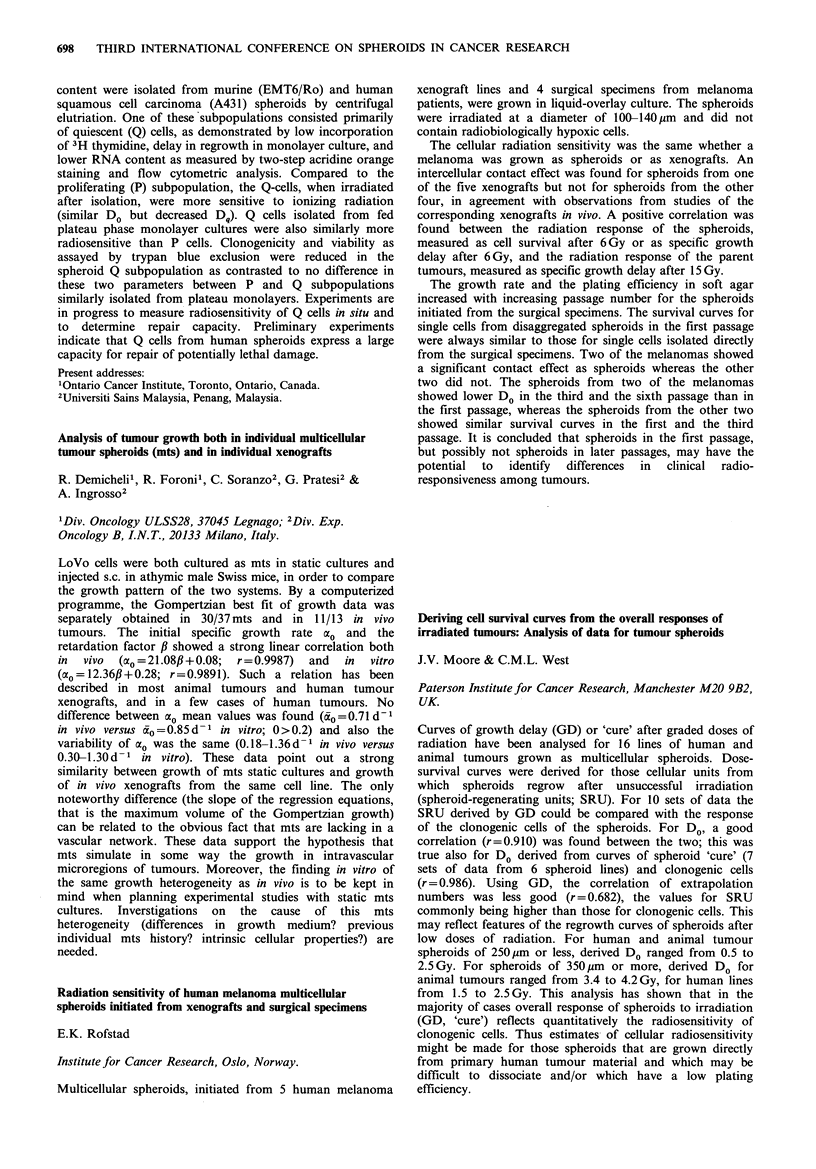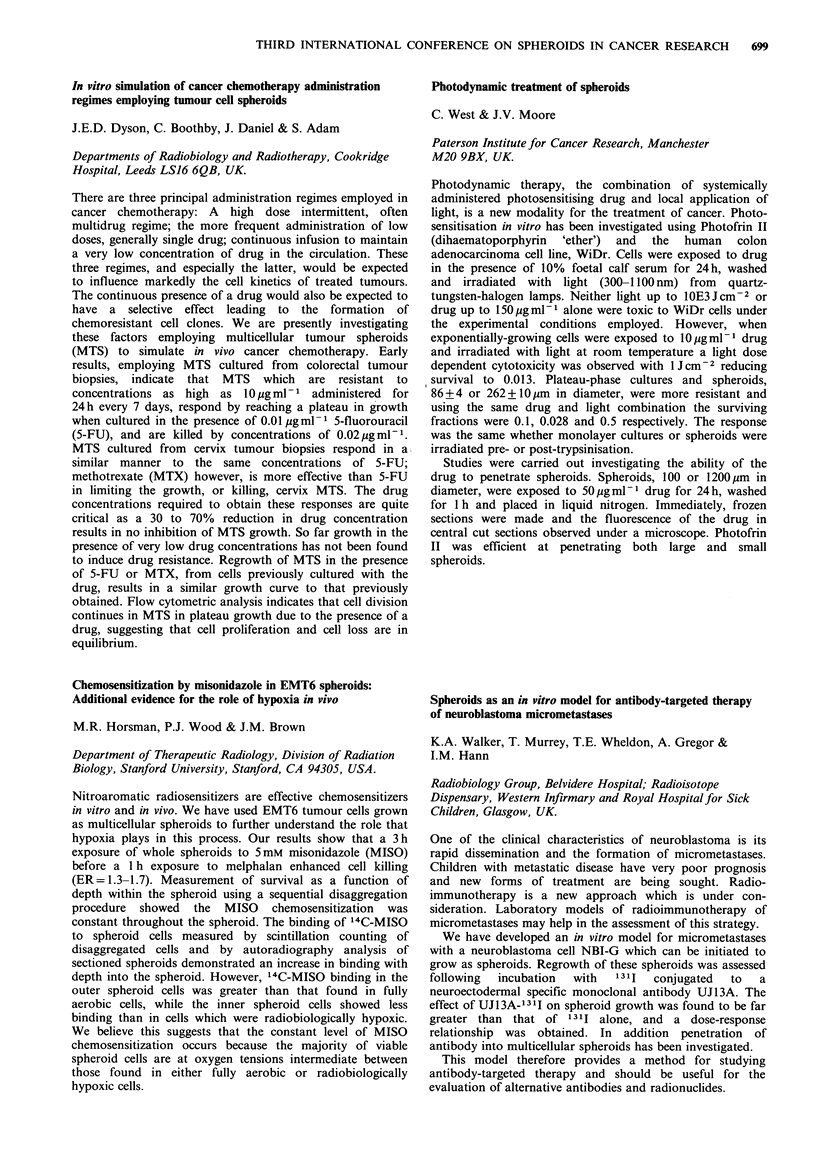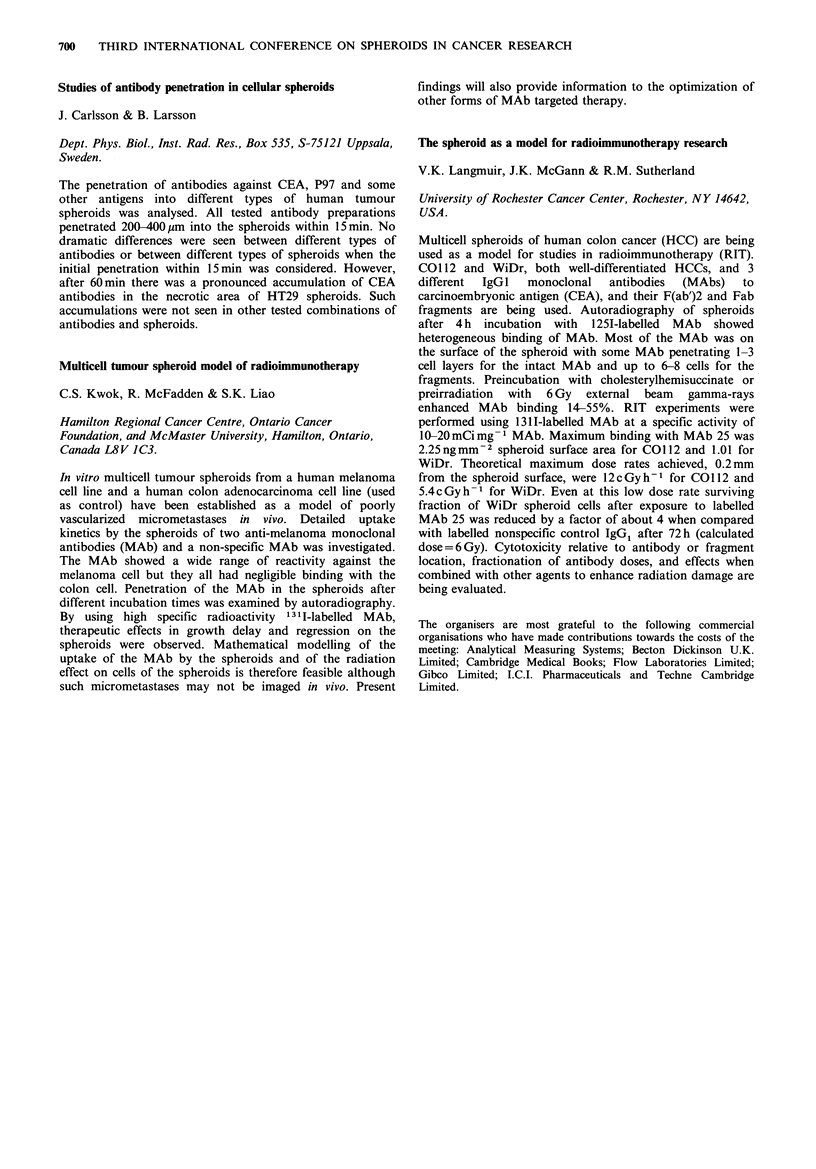# Third International Conference on Spheroids in Cancer Research (July 27-28, 1987). Cambridge, UK. Abstracts.

**DOI:** 10.1038/bjc.1987.268

**Published:** 1987-11

**Authors:** 


Br. J. Cancer (1987), 56, 691–700 © The Macmillan Press Ltd., 1987

### Third International Conference on Spheroids in Cancer Research* (July 27-28, 1987)

*Held at Sidney Sussex College, Cambridge, UK.*

### Abstracts of Invited and Proffered Papers

### Growth and differentiation of human tumour spheroids

R. Sutherland, L. Goldsmith, A. Lane, V. Langmuir, E. Rofstad & D. Penney

*University of Rochester Cancer Center, Rochester, NY 14642, USA.*

Availability of different human spheroid experimental models within specific histopathological types of tumours would be useful for studies of heterogeneity of phenotypic properties, including therapeutic resistance, associated with progression of malignancy. In addition to intrinsic cellular heterogeneity, modifications associated with differentiation or other consequences of three-dimensional growth affecting cellular interactions and microenvironments may occur. We have characterized the growth and differentiation of spheroids of human squamous cell carcinomas (SCC) from different tissues of origin, colon adenocarcinomas, and an ovarian carcinoma. Differentiation was evaluated morphologically, biochemically, and with monoclonal antibodies. Several lines of spheroids which express different degrees of differentiation have now been established. Some colon adenocarcinoma spheroids develop glandular-like structures and produce carcinoembryonic antigen (CEA) heterogeneously distributed throughout the spheroids. Antibodies to different forms of keratin and anchoring fibrils are expressed differentially in the different SCC spheroids. Evidence of squamous differentiation is also present morphologically in some of these. Morphological studies and antibody reactivities with ovarian carcinoma spheroids are currently in progress. These spheroids are being further characterized relative to their properties when grown as monolayers and xenograft tumours.

### Intercellular junctions and tumour cell invasion in multicellular spheroids

T. Bräuner & D.F. Hülser

*Abt. Biophysik, Biologisches Institut, Universität Stuttgart, Pfaffenwaldring 57, D-7000 Stuttgart 80, FRG.*

To answer the question whether the capacity of tumour cells to communicate with each other and with normal tissue via gap junctions has any influence on their invasive behaviour, we confronted chick heart fragments with multicellular tumour spheroids of 5 tumour cell lines, according to an *in vitro*-invasion assay developed by Mareel and coworkers. Mammary tumour cells of the rat (BICR/M1R-k) and mouse (EMT6/Ro) as well as rat glioma cells (C6) revealed gap junctions in ultrathin sections of freeze-fracture preparations. With electrophysiological techniques both electrical and dye coupling was demonstrated in these 3 cell lines. In co-cultures, all 3 communicating tumour cell lines also exhibited heterologous electrical coupling with normal tissue (embryonic chick heart cells). In contrast, HeLa cells (human cervix carcinoma), which are linked by tight junctions, and L-cells (mouse sarcoma), exhibiting desmosomes at regions of intercellular contact, have no gap junctions and are unable to communicate with each other or with normal embryonic cells. The coupling-competent tumour cell lines occupied and progressively replaced the heart tissue within 4 days. In contrast, the non-coupling HeLa cells destroyed the heart tissue much later and by a totally different mechanism, in which the epithelial organization of these cells seems to play a major role. The non-coupled mouse sarcoma cell line L only formed a solid capsule around the heart aggregate, without invading it.

### The use of spheroids and artifical tumours in the study of invasion and metastasis

E. Boghaert, G. De Bruyne & M. Mareel

*Laboratory of Experimental Cancerology, University Hospital, Ghent, Belgium.*

Invasion and metastasis are hallmarks of malignant tumours. Experimental systems for the study of malignancy are confronted with two problems: (i) since invasion implies by definition both the tumour cells and the host, the method to analyze invasion *in vitro* becomes more complicated than e.g. the study of growth; (ii) spontaneous tumours are 3-dimensional structures whereas cell cultures are monolayers or cell suspensions.

Invasion *in vitro* is analyzed by confrontation of spheroids of malignant cell populations with fragments of embryonic tissues in organ culture. Invasion and metastasis *in vivo* is tested by means of implantation of artifical tumours (cells attached to a collagen matrix) respectively underneath the renal capsule and underneath the skin of the tail in syngeneic animals. The relevance and reliability of the assays are checked by comparing invasive and non-invasive cell populations.

The implantation of artificial tumours underneath the renal capsule of syngeneic animals creates the possibility to evaluate quantitatively invasion of different cell lines (e.g. $MO_4$, RAC 11P, RAC 5E, B16B16) as a function of time. The former test is reproducible ($MO_4$) and specific for the tested cell line. Implantation of artificial tumours of B16B16 melanoma under the renal capsule and s.c. in the tail permits evaluation of the effect of 'site dependency' of malignancy. Confrontation of spheroids composed of mixtures and invasive and non-invasive cell populations (MCF-7 and HBL-100; RAC 11P and RAC 5E) with precultured heart fragments indicates possible influences of one cell population on the invasive behaviour of the other.

### Intercellular adhesivity in composite spheroids

C. Chauzy, B. Delpech, A. Olivier & N. Girard

*Laboratory of Immunochemistry, Centre Henri Bacquerel, 76000 Rouen, France.*

The co-culture of fibroblasts with cancerous cells under the

*Organisers: H. Acker, N.J. McNally, I.J. Stratford, R.M. Sutherland and P.R. Twentyman.

conditions which lead to spheroid formation yielded spheroids composed of a fibroblastic core surrounded by cancerous cells. The formation of a fibroblastic core was observed after starting co-culture of cells at various times: fibroblasts + cancer cells, fibroblast spheroids + cancer cells, cancer spheroids + fibroblasts, cancer spheroids + fibroblast spheroids. Therefore cancer cells have more affinity for fibroblasts than for other cancer cells. There was no species specificity in the cell–cell affinities. Human glioma, hepatoma, breast adenocarcinoma and others, and rat fibrosarcoma cells formed composite spheroids with human fibroblasts.

The extra cellular matrix was studied by antibodies against collagen type I, III, IV, fibronectin, hyaluronectin. Hyaluronic acid was studied by affinoimmunology. No qualitative difference was found between cancer spheroids and fibroblast spheroids.

Inhibition of spheroid formation was attempted by use of antibodies, GAG, proteins, carbohydrates, enzymes. Elastase inhibited the cancer spheroid formation and disaggregated cancer spheroids without cytotoxicity. Fibroblast spheroids were not affected by elastase. Other enzymes (hyaluronidase, collagenase) had no or had limited effect on both spheroids, whereas trypsin disaggregated both types. The results suggest that elastase plays a prominent role in cancer cell adhesivity *in vitro*.

### The mobility of plasma membrane lipids depends on cell-substrate and cell–cell contacts

M. Stuschke[1], R. Krieg[2], V. Budach, H. Bojar[2] & M. Molls[1]

[1]*Department of Radiotherapy, University of Essen, FRG.*
[2]*Department of Oncological Chemistry, University of Düsseldorf, FRG.*

Translational diffusion of fluorescent lipid probes in the plasma membrane of single, viable cells – either in mono-cellular suspension or attached on glass coverslips or integrated in the outer rim of a spheroid – was measured by the technique of fluorescence recovery after photo-bleaching. The cell line HMF-1, used here, was derived from a xenograft of a human high grade malignant fibrous histiocytoma at passage 8. The fluorescent lipid probe was 1-acyl-2-(N-4-nitrobenzo-2-oxa-1,3-diazole) aminocaproyl phosphatidylcholine (NBD-PC). At 21°C, the diffusion coefficients in the apical, medium facing plasma membrane of cells, growing on glass substrate and in the outer rim of spheroids of 1 mm diameter, were reduced by 45% ($P<0.0001$) and 64% ($P<0.0001$) respectively in comparison with cells in suspension. The mobile fraction of lipids in the membrane of suspended cells (61%) was higher than in spheroid integrated cells (35%) ($P<0.0001$). Measurements were also performed at 37°C, though a slight proportion of dye molecules diffused in the cytoplasm. At the higher temperature, the diffusion coefficients in spheroid integrated cells were reduced by 58% ($P<0.001$) compared to suspended cells but no effect on the mobile lipid fraction was seen.

It is concluded that cell-substrate and cell–cell contacts modulate plasma membrane lipid dynamics, which can regulate various membrane enzymes, transport processes, effector-membrane receptor interactions and the vertical displacement of membrane proteins.

### DNA-DNA crosslinks can explain the contact effect

P.L. Olive

*British Columbia Cancer Research Centre, Vancouver, Canada.*

Chinese hamster V79-171B lung fibroblasts grown for 20 h in suspension culture form clusters of cells which are more resistant to damage by ionizing radiation. DNA unwinding kinetics, measured using the alkali-unwinding assay, suggest that cells placed in suspension culture develop constraints to unwinding at the same rate as they develop resistance to the cytotoxic effects of radiation. In attempts to identify these constraints, we have concentrated on two likely candidates: DNA/protein and DNA/DNA crosslinks. DNA/protein crosslinks might occur if DNA binds to the nuclear protein matrix. However, rates of digestion of DNA from isolated nuclei or matrices were identical for monolayers and spheroids. Addition of 2% SDS or protease during denaturation had no influence on DNA unwinding rates of monolayer or spheroid cultures although labelling cells with $^3$H-leucine indicated that over 70% of the protein was digested with this treatment.

When cells are sonicated in alkali, most of the DNA becomes single-stranded but ~15% renatures upon subsequent neutralization. However, ~20% of the spheroid cell DNA was double-stranded after cells were sonicated in alkaline solutions. This result would be expected to occur if DNA/DNA crosslinks were present in spheroid DNA. Assuming that sonication breaks DNA into pieces about 4,000 bases long ($\sim 1.5\,\mu$), and crosslinks are present every $50\,\mu$, then one would expect that about 3% of the sonicated pieces would contain a crosslink. Crosslinked pieces would appear as double-stranded, and could thus explain the larger amount of DNA which renatures with spheroids. These results suggest that, for reasons that are not yet clear, DNA/DNA crosslinks may form in spheroid DNA and influence response to DNA damage.

### Correlation between gapjunctional communication and radioresistance in glioblastoma spheroids; influence of gapjunctional uncouplers

G. Knedlitschek, K.F. Weibezahn & H. Dertinger

*Kernforschungszentrum Karlsruhe, HS/Biophysik, POB 3640, D-7500 Karlsruhe, FRG.*

As already demonstrated in our laboratory, the so-called contact effect (CE) of various cell lines is correlated with the ability of cells to perform intercellular communication (IC) via gap junctions (GJ). Based on this correlation we were able to rank the spheroid radioresistance of 3 rat glioblastomas (RG2, F98 and 9L) according to their relative degree of IC as determined by microelectrode techniques. The mean killing dose and the degree of differentiation increased in correlation to IC (lowest in RG2, highest in 9L).

As an approach to study the biological role of GJ we are presently investigating the action of gapjunctional inhibitors on radiosensitivity and IC. We studied several gapjunctional uncouplers such as ouabain and the tumour promoter TPA upon survival of Chinese hamster peritoneal cells (line B14FAF28). Pretreatment with the drugs resulted in a sensitization against CE. The monolayer survival remained unaffected.

### Enhanced recovery from growth inhibition (RGI) in irradiated MO4 spheroids invading into embryonic chick heart fragments in organ culture

G. Storme[1] & M. Mareel[2]

[1]*Cancer Research Unit, Oncologic Center, Vrije Universiteit, Brussels, Belgium.*
[2]*Laboratory of Experimental Cancerology, Department of Radiotherapy and Nuclear Medicine, University Hospital, Ghent, Belgium.*

Growth of irradiated spheroids was followed in suspension cultures, after explanation on glass and after trypsinisation of irradiated spheroids by colony forming efficiency in a culture vessel. We found that MO4 spheroids recovered better from higher doses of ionizing irradiation (IR) when they were explanted on glass than while kept in suspension culture suggesting that the irradiated MO4 population became anchorage dependent for growth. However RGI after 18 Gy was significantly higher when MO4 spheroids confronted fragments of embryonic chick heart in suspension culture as compared to these explanted on glass in a Leighton tube ($P<0.006$) or to gel-form in suspension culture ($P<0.007$). These latter results suggest that the normal tissue might contribute to RGI in irradiated MO4 populations.

**Radiation response of human tumour spheroids**

R. Sutherland, T. Kwok, V. Langmuir, J. McGann & E. Rofstad

*University of Rochester Cancer Center, Rochester, NY 14642, USA.*

Spheroids of human colon carcinoma (HCC), squamous cell carcinoma (SCC), and ovarian carcinoma (OC) have been irradiated at different stages of growth. Poorly (HT29) and moderately (WiDr) differentiated HCC spheroids developed significant radiation resistant hypoxic fractions at diameters greater than 800 $\mu$M when grown in medium equilibrated with 20% $O_2$ with the spinner flask kept sealed for 24 h before irradiation. The well differentiated (CO112) HCC spheroids which contain glandular structures and necrotic centres had no resistant hypoxic cells or a much smaller hypoxic fraction at similar sizes compared with HT29 spheroids. This is consistent with previous measurements with microelectrodes of oxygenation in these spheroids. There was little or no contact effect to radiation in small spheroids of these HCC lines. CaSki SCC spheroids grown to maximum diameters of 500–600 $\mu$m did not contain resistant hypoxic cells despite the presence of central necrosis. However, the viable rim of cells in spheroids is unusually thin for this cell line. Small CaSki spheroids exhibited a contact effect. A431 spheroids (SCC) at 700–800 $\mu$m contained a small hypoxic but not maximally resistant cell fraction. A contact effect is not a frequent phenomenon in OC spheroids, but has been demonstrated in one line so far. Experiments are in progress to determine the frequency of the hypoxic fraction and the contact effect among these and other types of human tumour spheroids.

**New techniques for the sorting and selective dissociation of spheroids**

J.P. Freyer, P.L. Schor, M.E. Wilder & J.H. Jett

*Los Alamos National Laboratory, Mail Stop M880, Los Alamos, New Mexico 87545, USA*

We have developed a flow cytometric method for sorting viable, intact spheroids in order to obtain uniformly-sized populations with diameters in the range of 50–100 $\mu$m. Unstained, viable spheroids were simultaneously analyzed for forward-angle light scatter (FALS), 90° light scatter and autofluorescence. By setting narrow sort windows on the FALS signal and either of the other signals, uniformly-spherical populations of spheroids could be recovered with a 90–100% efficiency. The sorted populations had coefficients of variation in the range of 5–9%, representing a variation of less than one cell diameter; uniformity was maintained with further growth. Applications of this technique will be discussed, along with work under way to allow the flow analysis and sorting of larger spheroids.

We have also developed an automated apparatus to dissociate spheroids into subpopulations of cells from different locations in the cell rim. This device has several advantages over current techniques, including: dissociation of a large number of spheroids simultaneously (300 spheroids 1200 $\mu$m in diameter yields $\sim 10^7$ cells in each of 10–15 fractions); automated operation; rapid cell recovery (10–15 cell fractions in 30–45 minutes); and precise control over dissociation conditions, allowing easy adaptation to different cell lines. Importantly, the spheroids are not handled after being placed into the dissociation chamber; uniform trypsin exposure is maintained throughout the dissociation period. We will demonstrate the uniformity and reproducibility of this method and its application to spheroids of 5 different cell lines.

**Spheroids in the study of tumour immunity**

K.M. Wilson & E.M. Lord

*University of Rochester Cancer Center, Rochester, NY 14642, USA.*

The multicellular tumour spheroid (MTS) is a useful tumour model for the study of *in situ* immunity. A milder dissociation procedure than those used for solid tumours results in a high yield of viable host immune cells while preserving cell markers and functional activity. MTS can be grown serum-free to avoid nonspecific host cell activation.

Spheroids of EMT6/Ro have proven useful in the study of cells important in rejection at the tumour site (Wilson & Lord, *Br. J. Cancer* **55**, 141, 1987). We have shown that spheroid associated host immune cells from EMT6 immunized syngeneic BALB/c mice have a greater cytolytic activity against EMT6 than do host cells from other locations. The cell responsible for this activity is an antigen specific cytotoxic T lymphocyte. No natural killer cell activity was present in the infiltrating host cell population. However, there was cytolytic activity against WEHI-164 (a line sensitive to tumour necrosis factor) which was mediated by a macrophage population.

The MTS has also been useful in examining the effects of ionizing radiation on host-tumour interactions (Wilson & Lord, *Cancer Immunol. Immunother.* **23**, 20, 1986). Unlike solid tumours, host and tumour can be irradiated separately. We have shown that mature effector cytolytic activity is radiation resistant. When animals are irradiated earlier in the immune response to the tumour cells, there is a detrimental effect to the host in terms of decreased host cell numbers and activity even though some cytolytic effector cells are still present.

**Metabolic imaging in spheroids using bioluminescence**

S. Walenta & W. Mueller-Klieser

*Department of Applied Physiology, University of Mainz, D-6500 Mainz, FRG.*

The spatial distribution of glucose, lactate and ATP was determined in multicellular HT29 spheroids by a modified bioluminescence technique that was originally designed for the use in brain tissue (Paschen *et al.*, *J. Neurochem.* **36**, 513, 1981). For measurement, spheroids were rapidly frozen and subjected to serial sectioning in a cryostat. The substrates of interest were enzymatically linked to the bioluminescence of luciferase by placing a cryostat section of a frozen cocktail with appropriate enzymes on top of the frozen spheroid section. Photon emission which was initiated by thawing these sections was recorded by film exposure with subsequent evaluation by microdensitometry and image analysis. Distributions of the different substrates investigated could be registered in successive cryostat sections, i.e., at similar locations in the spheroid. Although the data were obtained on a relative scale so far, preliminary experiments show that the concentration profiles measured can be calibrated in absolute terms. The results obtained clearly show pronounced regional differences in all substances investigated. The distribution of ATP was correlated with the histological structure of the spheroids showing high values in the viable cell rim and low values close to or at the background level in the necrotic core of the spheroids. There was no indication of low glucose levels in the centre of these cell aggregates. Also, an obvious correlation among the parameters measured could not be found.

### High-resolution NMR imaging of conditions inside intact, viable spheroids

J.P. Freyer[1], L.O. Sillerud[1] & M. Mattingly[2]

[1]*Los Alamos National Laboratory, Mail Stop M880, Los Alamos, NM 87545;* [2]*Bruker Instruments, Billerica, MA 01821, USA.*

We have used high-resolution proton NMR imaging of intact spheroids to demonstrate the feasibility of making noninvasive measurements of morphology and microenvironmental conditions inside spheroids. Spheroids ($\sim 1500\,\mu m$ diameter) were placed in glass tubes and loaded into a special high-resolution NMR imaging system at Bruker Instruments. This system was adjusted to image $125\,\mu m$ thick sections through the intact spheroid, with a spatial resolution of 20–30 $\mu m$. By using multiplanar, multiecho imaging techniques, differences in the binding of water across the spheroid section were seen. The area of central necrosis bound water to a much greater extent than the area corresponding to viable cells, as indicated by the shorter spin–spin relaxation times ($T_2$). Computer processing of the data allowed a precise measurement of the extent of central necrosis in an intact spheroid which compared well to histological techniques. Preliminary phosphorous spectroscopy has shown that the metabolic energy status of cells in spheroids can be measured. Techniques are under development to enable the imaging of specific molecules, including glucose and lactate, which would then allow the measurement of nutrient concentration gradients inside viable spheroids. We have built a system for holding a viable spheroid in a perfused sample tube so that we can image conditions inside spheroids under the same conditions as those used for growth.

### Interrelationship among metabolic milieu, growth properties and oxygenation status of WiDr human colon carcinoma spheroids

M. Brach & W. Mueller-Klieser

*Department of Applied Physiology, University of Mainz, D-6500 Mainz, FRG.*

Multicellular spheroids of WiDr cells, an early passage human colon adenocarcinoma cell line, could be grown in suspension cultures up to maximum diameters of around 2.5 mm within 26 days. WiDr spheroids were cultured in media with 5.5 or 25 mM glucose or with 3 different lactate concentrations (3.5–20 mM) equilibrated with 20% or 5% $O_2$. The aggregates were assayed for volume growth, cellularity, histological structure, and oxygenation status as a function of time in culture. The oxygenation was quantified by a microelectrode technique published elsewhere (Mueller-Klieser & Sutherland, *Cancer Res.*, **42**, 237, 1982). An elevation of the external glucose concentration was associated with an increase in the thickness of the viable cell rim. Increased lactate concentrations in the culture medium had an adverse effect on cellular viability in these spheroids. Volume growth kinetics and cell content were influenced in a corresponding way. Oxygen tensions in WiDr spheroids were less than in EMT6 spheroids of similar sizes as measured previously. WiDr spheroids exhibited pseudoglandular structures within their viable cell rim and released carcinoembryonic antigen (CEA) into the culture medium. Preliminary data indicate that the CEA production in WiDr spheroids may be largely modified by the external growth conditions (courtesy of Dr B. Sordat).

### Glucose diffusion in multicellular spheroids

J.J. Casciari, S.V. Sotirchos & R.M. Sutherland

*University of Rochester, Chemical Engineering Department and Cancer Center, Rochester, NY 14642, USA.*

In order to understand spheroid microenvironment, knowledge of glucose transport is essential. The effective glucose diffusion coefficient has been measured in spheroids of both human and rodent tumour cell lines using tritium-labeled L-glucose as a diffusion probe. Values vary significantly with cell line: EMT/Ro spheroids have glucose diffusion coefficients of $1.05 \times 10^{-6}\,cm^2\,sec^{-1}$ while the diffusion coefficients of the human cell spheroids used (A431, CaSki, HT29, CO112, WiDr) range from $6 \times 10^{-7}\,cm^2\,sec^{-1}$ to $2 \times 10^{-7}\,cm^2\,sec^{-1}$. In cases where the diffusion coefficient and the glucose consumption rate are both known, a mathematical model of nutrient transport has been used to calculate glucose concentration profiles in spheroids. The predictions of this model, based on diffusion coefficient values obtained in this study, can be used to assess the role of limiting glucose supply in spheroid growth and in the development of central necrosis. The model can also be used to calculate gradients in oxygen concentration, carbon dioxide concentration, lactate concentration, and pH that develop as spheroids grow.

### Acid pH as a potential cause of cell death in spheroids and tumours

I.F. Tannock, D. Steele-Norwood, D. Rotin & I. Kopelyan

*Ontario Cancer Institute, Toronto, Canada.*

We have reported previously that the combination of hypoxia and low pH ($\sim$pH 6.0) is very toxic to single cells in tissue culture, whereas neither condition alone causes rapid cell death. A probable mechanism is failure of cells to regulate their intracellular pH ($pH_i$) under hypoxic conditions. To determine whether $pH_i$ regulation is a determinant of cell viability in spheroids and tumours, we have selected mutant cells from the MGH-U1 human bladder cancer cell line which lack the $Na^+/H^+$ membrane ion exchanger, one of the major mechanisms for controlling $pH_i$. Mutant cells are very sensitive to acid pH in tissue culture; they grow only at pH 7.0 or above, whereas parental cells grow at pH 6.8. When implanted into immune-deprived mice, mutant cells either fail to grow or form very slowly-growing tumours, whereas tumours grow rapidly from wild-type cells. These findings suggest that the $Na^+/H^+$ exchanger is essential for tumour growth, and that cells lacking it may be unable to survive in the acidic environment which develops as the tumour grows. We are attempting to grow spheriods from the mutant cells, and will compare spheroid growth and formation of necrosis with that in spheroids derived from wild-type cells. Such experiments should determine the importance of regulation of $pH_i$ to spheroid growth and to the formulation of necrosis in their centre.

### Regulation of $pO_2$ and pH in cellular spheroids

H. Acker[1] & J. Carlsson[2]

[1]*Max-Planck Institut für Systemphysiologie, 4600 Dortmund, FRG;* [2]*Institute for Radiation Sciences, Uppsala, Sweden.*

Cellular spheroids of human and rodent origin exhibit simultaneously a $pO_2$- as well as a pH-gradient, indicating an aerobic glycolysis. $pO_2$-gradient as an expression of oxygen consumption and pH-gradient as an expression of lactate production are interdependent. Lowering the glucose supply results in a reduction of the pH-gradient and an increase of the oxygen consumption. Lowering the oxygen supply results in stepwise reduction of the respiratory chain and a further acidification of the tissue caused by an activation of the lactate dehydrogenase. Under these circumstances a higher glucose supply is demanded. The importance of this regulation can be demonstrated by the strong correlation between the growth rate of the spheroids in the exponential phase and the quotient $pO_2$-gradient/pH-gradient, showing that fast growing spheroids exhibit a higher oxygen consumption whereas slow growing spheroids exhibit a higher lactate production.

### Hypoxia and oxygen consumption in EMT6/ED spheroids

A.J. Franko & C.J. Koch

*Cross Cancer Institute, Edmonton, Alberta, Canada TG6 1Z2.*

A very complex relationship exists between oxygen and glucose concentrations, their rates of consumption, and cell death in hypoxia as demonstrated in studies of EMT6/Ro spheroids grown in BME. Our studies of EMT6/Ed spheroids grown in Waymouth's generally show a relatively simpler control of cell death, with few of the effects reported for EMT6/Ro. Over a very wide range of glucose concentrations, 0.3 to $5\,g\,l^{-1}$, we see no difference in the fraction of hypoxic cells as assessed by radiation survival curves. *In situ* oxygen consumption rates are identical as indicated by misonidazole binding patterns. Direct measurements of oxygen consumption rates of intact spheroids and cells isolated from spheroids are little different from those of exponentially growing monolayer cells, after correction for cell size. The thickness of the viable rim is reduced appreciably at $1\,g\,l^{-1}$ glucose or less and the transition between necrotic and intact cells becomes much more abrupt. The excess cells at high glucose, all of which are in the hypoxic region, are unable to survive disaggregation to contribute to radiation survival, although they are able to consume oxygen if the oxygen concentration in the medium is increased. EMT6/Ed spheroids grow more slowly in BME than in Waymouth's, at $1\,g\,l^{-1}$ glucose, and the radiobiologically hypoxic fraction is reduced in BME. Further studies of this difference are in progress. The ultimate cause of cell death is still unclear, even for the less complex situation of EMT6/Ed spheroids in Waymouth's. One difficulty is that the lifetime of hypoxic cells is approximately one day in normally growing spheroids, whereas when growth is arrested by a large dose of radiation, the surviving hypoxic cells remain clonogenic and hypoxic for several days.

### Mathematical models for $PO_2$ and pH profiles in cellular spheroids

J. Hilsmann, J. Wiesecke, T. Küpper & H. Acker

*Max-Planck Institut für Systemphysiologie, 4600 Dortmund, FRG.*

Cellular spheroids of different origin exhibit $PO_2$ and pH profiles, indicating a simultaneous oxygen consumption and lactate production. In this context mathematical models should be helpful to get a deeper understanding of interconnections between these two metabolic pathways. For this purpose mathematical models have to take into account areas with $PO_2$ values of zero Torr and with necrotic cells. Therefore, we used free boundary problems to recalculate from $PO_2$ profiles oxygen consumption values. Especially spheroids with steep $PO_2$ gradients reaching zero $PO_2$ values in the centre showed a $PO_2$-dependent oxygen consumption, whereas $PO_2$ profiles with high central $PO_2$ values could be recalculated with a constant oxygen consumption. Mathematical procedures have been developed to take into account asymmetric configuration and asymmetric oxygen supply of spheroids. Furthermore, the interdependence of local pH and local oxygen consumption as well as local $pO_2$ and local lactate production were considered in a mathematical model.

### The sandwich system: Misonidazole studies

L. Hlatky, R. Sachs & L. Alpen

*Biology and Medicine Division, University of California at Berkeley, CA 94720, USA.*

The sandwich system was developed to supplement the spheroid system as a tumour analog. Like a spheroid, a sandwich is a diffusion-limited multicellular system which develops a necrotic centre and viable border; within the viable border are spatial gradients of nutrients and metabolites; these induce gradients of cellular kinetic behaviour, morphology and ultrastructure. Differences between sandwiches and spheroids include that in a sandwich there is no 3-dimensional cell–cell contact and live cells are continuously viewable as the system heterogeneity develops. Thus the sandwich system should be a useful complementary system to spheroids in various investigations, including the study of drug diffusion, drug uptake and differential drug action on cells in heterogeneous environments and kinetic states.

To investigate the system's potential with regard to interactive drug studies and to characterize sandwich hypoxic regions we labelled sandwiches with $^3$H-misonidazole. Location of cells within a sandwich was preserved by fixation and MISO binding was assessed by autoradiography. Heavy binding was seen in all cells bordering the necrotic centre, with a greater than 50-fold difference between cells in the innermost, hypoxic region *versus* the outer, oxygenated region. The misonidazole binding profiles were, at least approximately, consistent with previously computed oxygen profiles. It was possible to count grains per cell rather than grains per unit area.

Supplementary viability tests showed that heavily labelled cells are viable if restored to favourable growth conditions.

### Drug interactions in V79 spheroids

R.E. Durand

*B.C. Cancer Research Centre, Vancouver, B.C., Canada.*

The spherical symmetry and tissue-like nature of V79

spheroids make the model system attractive for the study of single or multidrug effects, and their modification by micro-environmental changes. We have had a particular interest in 'chemosensitization' using both hypoxic cell radiosensitizers and more conventional anti-neoplastic drugs as potentiators. By intercomparing drug distribution (using fluorescent or radioactively-labelled probes) with cytotoxicity, the viability of separable sub-populations within the spheroids can be related to cell location and thus physiological status. Additionally, use of the 'Median-Effect' analysis for drug–drug interactions allows (simultaneous) quantitative estimates of interaction as a function of drug concentration, cell survival level, and cell position in the spheroid. Examples will be presented for AF-2 and MISO potentiation of CCNU toxicity, and VP-16 potentiation of *cis*-platinum.

### A tritiated thymidine suicide method for the study of drug response of cells located at different depths within spheroids

T.T. Kwok and P.R. Twentyman

*MRC Clinical Oncology and Radiotherapeutics Unit, MRC Centre, Hills Road, Cambridge CB2 2QH, UK.*

A technique using 'tritiated thymidine suicide' has been established as a means of studying the response to cytotoxic drugs of cells at different depths within multicellular tumour spheroids. Because of the characteristic spatial arrangement of cycling cells (mostly in the outer regions) and non-cycling cells (mostly at the inner regions) of spheroids, cells surviving after long term (24 h) exposure of spheroids to high doses of $^{3}$HTdR will be those located furthest from the surface. By comparing the drug response of cells from $^{3}$HTdR pretreated and untreated spheroids, the individual response of total cells, cells near to the surface and cells lying deeper within the viable rim of spheroids can therefore be deduced. In this study, large spheroids of about 800 $\mu$m in diameter of a mouse mammary cell line, EMT6/Ca/VJAC, and of a human small cell lung cancer cell line, POC, have been used. We have confirmed that (1) the cells killed are those which incorporate $^{3}$HTdR during the synthesis period; (2) the cells killed are mainly located in the outer regions of spheroids, i.e. surviving cells are mostly located in the inner part of the viable rim; and (3) $^{3}$HTdR pretreatment does not sensitise surviving cells to subsequent cytotoxic drug treatment. Results from large EMT6 spheroids agree with our previous findings (obtained using a selective dis-aggregation method) that cells in the outer regions of spheroids are more sensitive to ADM and HN2 than cells in the inner regions whilst the opposite is true for CCNU. For POC spheroids, cells in the outer region of spheroids are more sensitive to ADM and VCR than cells in the inner region whilst a reverse trend is seen for the response to CCNU. The response to HN2 is similar at all depths.

### Spheroids for testing formulated drugs: VP-16 (etoposide)

J.M. Cook[1], D. Kerr[2], T. Wheldon[3] & A.T. Florence[1]

[1]*Department of Pharmacy, University of Strathclyde;* [2]*Department of Medical Oncology, Glasgow University;* [3]*Department of Radiotherapeutics, Belvidere Hospital, Glasgow, UK.*

VP-16, a drug of low aqueous solubility (80 $\mu$g ml$^{-1}$), is formulated for intravenous and oral use as a solubilised preparation. The vehicle contains a surfactant (polysorbate 80), polyoxyethylene glycol 300 and ethanol. We have been studying the influence of surfactant on the penetration of drugs into tissues and the effect of drug precipitation from solubilised preparations *in vivo* on bioavailability. Human non-small cell lung cancer, L-Dan, and human neuro-blastoma, $NB_1$ spheroids have been used to examine the penetration and activity of formulated and unformulated drug, by autoradiography, analysis of stripped layers and growth delay measurements. The last suggest that the drug is equipotent in its two forms up to 60 $\mu$g ml$^{-1}$, the superiority of the formulated material thereafter being due to the increased levels of drug in the solution state, and the penetration enhancing properties of the surfactant. Surfactants Brij 58 (a polyoxyethylene cetyl ether) and Triton X-100 (a polyoxyethylene nonylphenyl ether) cause rapid swelling of spheroids at 1 mg ml$^{-1}$, while polysorbate 80 has no such effect. This swelling indicates penetration of the surfactant into the spheroid leading to disruption, an effect which may not have therapeutic significance but which limits the usefulness of the spheroid system. However such effects might be useful in determining the biological activity of surfactant molecules, which is a function of both penetration and intrinsic activity.

### Penetration of four different types of cytostatics into human glioma U-118MG and human colon carcinoma HT29 multicellular spheroids

M. Erlansson[1] & J. Carlsson[2]

[1]*Department of Oncology, University of Umeå, Umeå, Sweden;* [2]*Department of Physical Biology, Institute of Radiation Research, Box 531, Uppsala, Sweden.*

The penetration, binding and uptake of 4 different cytotoxic drugs (actinomycin D, adriamycin, daunomycin and cytosine arabinoside) was analysed in 2 types of cellular spheroids (glioma U-118MG and colon carcinoma HT29). All drugs penetrated rather well into the U-118MG spheroids. The intercalating drugs (actinomycin D, adriamycin and daunomycin) had a heterogeneous uptake corresponding to intercalation in cell-nuclei. Cytosine arabinoside was more homogeneously distributed. Actinomycin D and adriamycin penetrated also well in HT29 spheroids. The intercalation pattern was not so pronounced for the HT29 spheroids as for the U-118MG spheroids. A marked penetration gradient was seen for daunomycin in HT29 spheroids even after incubation times of one hour. Thus, HT29 spheroids have other properties than U-118MG spheroids when penetration is considered. This was especially so when the penetration of cytosine arabinoside was considered. The HT29 spheroids had in this case an efficient penetration barrier and all cytosine arabinoside seemed to be bound to the glycocalix matrix outside the spheroids. The binding persisted washing in medium without drug.

### Intravesical chemotherapy: Drug sensitivities of monolayers and spheroids evaluated by clonogenic cell survival, proliferation pattern and ultrastructure

R. Knüchel[1], F. Hofstäder[1], W. Jenkins[2] & J. Masters[2]

[1]*Department of Pathology, RWTH Aachen, FRG;* [2]*Institute of Urology, London WC2 9AE, UK.*

To assess *in vitro* the importance of drug penetration during intravesical chemotherapy, MCTS and monolayers of the human continuous bladder cancer cell line MGH-U1 were exposed for 1 h to adriamycin, epirubicin, epodyl, mitomycin-c or thiotepa. Monolayers growing exponentially on tissue culture plastic (Falcon) and MCTS propagated in microcarrier stirrers (Techne) following inoculation of $500 \times 10^{6}$ cells in 500 ml supplemented RPMI 1640 medium were used. Clonogenic assays showed that thiotepa was unique in being more cytotoxic in three than in twodimensional culture. MGH-U1 cells as MCTS were more resistant to epirubicin than mitomycin-C, the reverse of the results in monolayer culture. Bromodeoxyuridine (BrdU)-anti BrdU staining of cells synthesising DNA was used to measure the cytostatic effect 24 and 72 h after a 1 h exposure. Equitoxic concentrations of adriamycin and thiotepa produced different proliferation patterns, reflecting differences in their ability to penetrate MCTS. The proportion of proliferating cells was quantified using an automated image analysis system (LEITZ TAS). The differences in penetration were confirmed by ultrastructural studies of the drug-treated MCTS. In conclusion, these *in vitro* findings are consistent with clinical observations. MCTS are a useful model system with which to study drugs used for topical chemotherapy.

**The accumulation and toxicity of anthracyclines in multicell spheroids and in monolayers**

T.J. Bichay, W.R. Inch, E.G. Adams, J.E. Brewer, W.J. Adams & B.K. Bhuyan

*Department of Biophysics, University of Western Ontario, The London Regional Cancer Centre, London, Ontario N6A 4G5, Canada; and The Department of Cancer and Viral Diseases Research, The Upjohn Company, Kalamazoo, Michigan, USA.*

We have used a combination of HPLC, radiolabel, and flow cytometric techniques to measure the uptake of three anthracyclines: mitoxantrone, menogaril and adriamycin, in V79-OCF4 cells grown as monolayers, as 100 $\mu$m multicell spheroids, and as 650 $\mu$m spheroids. The 650 $\mu$m spheroids were dissociated into two fractions comprising an outer cell layer (50 $\mu$m thick) and an inner cell layer (100 $\mu$m thick). The toxicity of each of the three anthracyclines on the V79 cells grown in the various culture conditions was normalized to drug accumulation within the cells. The results demonstrated a reduced uptake of drug in the spheroids compared to the monoloayers. Anthracycline uptake was 5 times lower compared to monolayers. The LD90 of spheroids exposed to the anthracyclines was between 6 and 250 fold higher than in monolayers. However, once normalized for drug uptake, the LD90 was ~4 to 40 times greater for spheroids compared to monolayers. The small spheroids exhibit an intermediate sensitivity between that of monolayers and large spheroids. Cell subpopulations removed from the outer layer of the spheroid, are equisensitive to cells from the hypoxic inner layer for the three anthracyclines. The data suggest that restricted drug penetration or accumulation by cells in multicell spheroids only partially explains their resistance to anthracyclines.

**Fractionated treatment of human brain tumour spheroids**

D.F. Deen & L.E. Kendall

*Brain Tumor Research Center, University of California, San Francisco, CA 94143-0520, USA.*

In order to study the effects of fractionated X-ray treatment on spheroids, we used a standard protocol in which spheroids received from 1 to 30 fractions of X rays over a 15 day period and then were disaggregated and assayed for colony forming efficiency. Iso-effect curves for 3 human brain tumour cell lines (87 MG, 251 MG and 373 MG) were produced by plotting the total dose required to reduce survival to the 10% level *versus* the fraction number. All 3 curves were similar; to produce 90% cell kill, single doses of ~4 Gy were required, while total doses of ~8 Gy were required when the radiation was given in 8 fractions. The curves plateaued for fraction numbers >8. The hypoxic fractions of 251 MG and 373 MG cell lines were small, being $\leqq$5% for 600 $\mu$m diameter spheroids. Preliminary studies using SR2508 suggest that few, if any, hypoxic cells were in the 87 MG spheroids, because similar radiation survival curves were obtained in the presence and absence of this hypoxic cell sensitizer. Finally, intracellular GSH levels varied considerably among the spheroid types, but were relatively independent of spheroid size. We are hopeful that studies on these and other human brain tumour spheroids will help to define the major determinants of sensitivity to fractionated radiation treatment.

**The effect of different schedules of fractionated radiation on human neuroblastoma spheroids**

I. Berry, T.E. Wheldon, J.A. O'Donoghue, A. Gregor & I.M. Hann

*Radiobiology Group, Belvidere Hospital, Glasgow G31 4PG, UK.*

Hyperfractionation (multiple small doses) has been suggested as an advantageous strategy in radiotherapy. A special case is hyperfractionated total body irradiation (TBI) and bone marrow rescue for treatment of neuroblastoma micrometastases. Spheroids provide a realistic *in vitro* model of micrometastases and can be used to test this strategy. Human neuroblastoma lines NBI-G and IMR-32 were grown as spheroids and subjected to a variety of radiation regimes which had been calculated to be isoeffective for damage to late-responding normal tissues (as assessed by the Linear Quadratic model with $\alpha/\beta = 3$ Gy). Spheroid response to irradiation was evaluated as regrowth delay or 'proportion cured'. The results show hyperfractionation to be a superior strategy for NBI-G but the advantage is less marked for IMR-32. Spheroids provide a useful model for testing fractionation strategies in radiotherapy.

**Influence of ionizing radiation on growth and oxygen profiles of different types of multicellular spheroids**

T. Nylén[1], J. Carlsson[2], G. Holterman[3] & H. Acker[3]

[1]*Dept. Rad. Biol., National Def. Res. Inst., S-90182 Umeå,* [2]*Dept. Phys. Biol., Inst. Rad. Res., Box 535, S-75121 Uppsala, Sweden and* [3]*Max-Planck Inst. Systemphysiol., Rheinlanddamm 201, 4600 Dortmund, FRG.*

Different types of cell-spheroids were irradiated with 137-Cs to doses typical in tumour therapy. Disturbances in the growth patterns were analysed from growth curves and from incorporation of thymidine. Some interesting variations in radiosensitivity were found. For example, the colorectal carcinoma HT-29 spheroids were very sensitive showing degeneration after moderate doses while the glioma U-118MG spheroids were more resistant. Oxygen gradients were measured with microelectrodes different times after irradiation. The oxygen gradients were not significantly changed the first 10 days after doses which gave severe growth disturbances. After longer times sometimes a small reoxygenation could be seen in parallel to radiation induced cell degeneration.

**Identification and radiosensitivity of quiescent and proliferating subpopulations in multicellular spheroids**

C. Luk[1], P. Keng, C. Ng[2] & R. Sutherland

*University of Rochester Cancer Center, Rochester, NY, USA.*

Two subpopulations enriched in cells with a $G_1$-like DNA

content were isolated from murine (EMT6/Ro) and human squamous cell carcinoma (A431) spheroids by centrifugal elutriation. One of these subpopulations consisted primarily of quiescent (Q) cells, as demonstrated by low incorporation of $^3H$ thymidine, delay in regrowth in monolayer culture, and lower RNA content as measured by two-step acridine orange staining and flow cytometric analysis. Compared to the proliferating (P) subpopulation, the Q-cells, when irradiated after isolation, were more sensitive to ionizing radiation (similar $D_0$ but decreased $D_q$). Q cells isolated from fed plateau phase monolayer cultures were also similarly more radiosensitive than P cells. Clonogenicity and viability as assayed by trypan blue exclusion were reduced in the spheroid Q subpopulation as contrasted to no difference in these two parameters between P and Q subpopulations similarly isolated from plateau monolayers. Experiments are in progress to measure radiosensitivity of Q cells *in situ* and to determine repair capacity. Preliminary experiments indicate that Q cells from human spheroids express a large capacity for repair of potentially lethal damage.

Present addresses:
[1]Ontario Cancer Institute, Toronto, Ontario, Canada.
[2]Universiti Sains Malaysia, Penang, Malaysia.

### Analysis of tumour growth both in individual multicellular tumour spheroids (mts) and in individual xenografts

R. Demicheli[1], R. Foroni[1], C. Soranzo[2], G. Pratesi[2] & A. Ingrosso[2]

[1]*Div. Oncology ULSS28, 37045 Legnago;* [2]*Div. Exp. Oncology B, I.N.T., 20133 Milano, Italy.*

LoVo cells were both cultured as mts in static cultures and injected s.c. in athymic male Swiss mice, in order to compare the growth pattern of the two systems. By a computerized programme, the Gompertzian best fit of growth data was separately obtained in 30/37 mts and in 11/13 *in vivo* tumours. The initial specific growth rate $\alpha_0$ and the retardation factor $\beta$ showed a strong linear correlation both *in vivo* ($\alpha_0=21.08\beta+0.08$; $r=0.9987$) and *in vitro* ($\alpha_0=12.36\beta+0.28$; $r=0.9891$). Such a relation has been described in most animal tumours and human tumour xenografts, and in a few cases of human tumours. No difference between $\alpha_0$ mean values was found ($\bar{\alpha}_0=0.71\,d^{-1}$ *in vivo versus* $\bar{\alpha}_0=0.85\,d^{-1}$ *in vitro*; $0>0.2$) and also the variability of $\alpha_0$ was the same ($0.18$–$1.36\,d^{-1}$ *in vivo versus* $0.30$–$1.30\,d^{-1}$ *in vitro*). These data point out a strong similarity between growth of mts static cultures and growth of *in vivo* xenografts from the same cell line. The only noteworthy difference (the slope of the regression equations, that is the maximum volume of the Gompertzian growth) can be related to the obvious fact that mts are lacking in a vascular network. These data support the hypothesis that mts simulate in some way the growth in intravascular microregions of tumours. Moreover, the finding *in vitro* of the same growth heterogeneity as *in vivo* is to be kept in mind when planning experimental studies with static mts cultures. Inverstigations on the cause of this mts heterogeneity (differences in growth medium? previous individual mts history? intrinsic cellular properties?) are needed.

### Radiation sensitivity of human melanoma multicellular spheroids initiated from xenografts and surgical specimens

E.K. Rofstad

*Institute for Cancer Research, Oslo, Norway.*

Multicellular spheroids, initiated from 5 human melanoma xenograft lines and 4 surgical specimens from melanoma patients, were grown in liquid-overlay culture. The spheroids were irradiated at a diameter of 100–140 μm and did not contain radiobiologically hypoxic cells.

The cellular radiation sensitivity was the same whether a melanoma was grown as spheroids or as xenografts. An intercellular contact effect was found for spheroids from one of the five xenografts but not for spheroids from the other four, in agreement with observations from studies of the corresponding xenografts *in vivo*. A positive correlation was found between the radiation response of the spheroids, measured as cell survival after 6 Gy or as specific growth delay after 6 Gy, and the radiation response of the parent tumours, measured as specific growth delay after 15 Gy.

The growth rate and the plating efficiency in soft agar increased with increasing passage number for the spheroids initiated from the surgical specimens. The survival curves for single cells from disaggregated spheroids in the first passage were always similar to those for single cells isolated directly from the surgical specimens. Two of the melanomas showed a significant contact effect as spheroids whereas the other two did not. The spheroids from two of the melanomas showed lower $D_0$ in the third and the sixth passage than in the first passage, whereas the spheroids from the other two showed similar survival curves in the first and the third passage. It is concluded that spheroids in the first passage, but possibly not spheroids in later passages, may have the potential to identify differences in clinical radio-responsiveness among tumours.

### Deriving cell survival curves from the overall responses of irradiated tumours: Analysis of data for tumour spheroids

J.V. Moore & C.M.L. West

*Paterson Institute for Cancer Research, Manchester M20 9B2, UK.*

Curves of growth delay (GD) or 'cure' after graded doses of radiation have been analysed for 16 lines of human and animal tumours grown as multicellular spheroids. Dose-survival curves were derived for those cellular units from which spheroids regrow after unsuccessful irradiation (spheroid-regenerating units; SRU). For 10 sets of data the SRU derived by GD could be compared with the response of the clonogenic cells of the spheroids. For $D_0$, a good correlation ($r=0.910$) was found between the two; this was true also for $D_0$ derived from curves of spheroid 'cure' (7 sets of data from 6 spheroid lines) and clonogenic cells ($r=0.986$). Using GD, the correlation of extrapolation numbers was less good ($r=0.682$), the values for SRU commonly being higher than those for clonogenic cells. This may reflect features of the regrowth curves of spheroids after low doses of radiation. For human and animal tumour spheroids of 250 μm or less, derived $D_0$ ranged from 0.5 to 2.5 Gy. For spheroids of 350 μm or more, derived $D_0$ for animal tumours ranged from 3.4 to 4.2 Gy, for human lines from 1.5 to 2.5 Gy. This analysis has shown that in the majority of cases overall response of spheroids to irradiation (GD, 'cure') reflects quantitatively the radiosensitivity of clonogenic cells. Thus estimates of cellular radiosensitivity might be made for those spheroids that are grown directly from primary human tumour material and which may be difficult to dissociate and/or which have a low plating efficiency.

### *In vitro* simulation of cancer chemotherapy administration regimes employing tumour cell spheroids

J.E.D. Dyson, C. Boothby, J. Daniel & S. Adam

*Departments of Radiobiology and Radiotherapy, Cookridge Hospital, Leeds LS16 6QB, UK.*

There are three principal administration regimes employed in cancer chemotherapy: A high dose intermittent, often multidrug regime; the more frequent administration of low doses, generally single drug; continuous infusion to maintain a very low concentration of drug in the circulation. These three regimes, and especially the latter, would be expected to influence markedly the cell kinetics of treated tumours. The continuous presence of a drug would also be expected to have a selective effect leading to the formation of chemoresistant cell clones. We are presently investigating these factors employing multicellular tumour spheroids (MTS) to simulate *in vivo* cancer chemotherapy. Early results, employing MTS cultured from colorectal tumour biopsies, indicate that MTS which are resistant to concentrations as high as $10\,\mu g\,ml^{-1}$ administered for 24 h every 7 days, respond by reaching a plateau in growth when cultured in the presence of $0.01\,\mu g\,ml^{-1}$ 5-fluorouracil (5-FU), and are killed by concentrations of $0.02\,\mu g\,ml^{-1}$. MTS cultured from cervix tumour biopsies respond in a similar manner to the same concentrations of 5-FU; methotrexate (MTX) however, is more effective than 5-FU in limiting the growth, or killing, cervix MTS. The drug concentrations required to obtain these responses are quite critical as a 30 to 70% reduction in drug concentration results in no inhibition of MTS growth. So far growth in the presence of very low drug concentrations has not been found to induce drug resistance. Regrowth of MTS in the presence of 5-FU or MTX, from cells previously cultured with the drug, results in a similar growth curve to that previously obtained. Flow cytometric analysis indicates that cell division continues in MTS in plateau growth due to the presence of a drug, suggesting that cell proliferation and cell loss are in equilibrium.

### Photodynamic treatment of spheroids

C. West & J.V. Moore

*Paterson Institute for Cancer Research, Manchester M20 9BX, UK.*

Photodynamic therapy, the combination of systemically administered photosensitising drug and local application of light, is a new modality for the treatment of cancer. Photosensitisation *in vitro* has been investigated using Photofrin II (dihaematoporphyrin 'ether') and the human colon adenocarcinoma cell line, WiDr. Cells were exposed to drug in the presence of 10% foetal calf serum for 24 h, washed and irradiated with light (300–1100 nm) from quartz-tungsten-halogen lamps. Neither light up to $10E3\,J\,cm^{-2}$ or drug up to $150\,\mu g\,ml^{-1}$ alone were toxic to WiDr cells under the experimental conditions employed. However, when exponentially-growing cells were exposed to $10\,\mu g\,ml^{-1}$ drug and irradiated with light at room temperature a light dose dependent cytotoxicity was observed with $1\,J\,cm^{-2}$ reducing survival to 0.013. Plateau-phase cultures and spheroids, $86\pm4$ or $262\pm10\,\mu m$ in diameter, were more resistant and using the same drug and light combination the surviving fractions were 0.1, 0.028 and 0.5 respectively. The response was the same whether monolayer cultures or spheroids were irradiated pre- or post-trypsinisation.

Studies were carried out investigating the ability of the drug to penetrate spheroids. Spheroids, 100 or $1200\,\mu m$ in diameter, were exposed to $50\,\mu g\,ml^{-1}$ drug for 24 h, washed for 1 h and placed in liquid nitrogen. Immediately, frozen sections were made and the fluorescence of the drug in central cut sections observed under a microscope. Photofrin II was efficient at penetrating both large and small spheroids.

### Chemosensitization by misonidazole in EMT6 spheroids: Additional evidence for the role of hypoxia *in vivo*

M.R. Horsman, P.J. Wood & J.M. Brown

*Department of Therapeutic Radiology, Division of Radiation Biology, Stanford University, Stanford, CA 94305, USA.*

Nitroaromatic radiosensitizers are effective chemosensitizers *in vitro* and *in vivo*. We have used EMT6 tumour cells grown as multicellular spheroids to further understand the role that hypoxia plays in this process. Our results show that a 3 h exposure of whole spheroids to 5 mM misonidazole (MISO) before a 1 h exposure to melphalan enhanced cell killing (ER = 1.3–1.7). Measurement of survival as a function of depth within the spheroid using a sequential disaggregation procedure showed the MISO chemosensitization was constant throughout the spheroid. The binding of $^{14}C$-MISO to spheroid cells measured by scintillation counting of disaggregated cells and by autoradiography analysis of sectioned spheroids demonstrated an increase in binding with depth into the spheroid. However, $^{14}C$-MISO binding in the outer spheroid cells was greater than that found in fully aerobic cells, while the inner spheroid cells showed less binding than in cells which were radiobiologically hypoxic. We believe this suggests that the constant level of MISO chemosensitization occurs because the majority of viable spheroid cells are at oxygen tensions intermediate between those found in either fully aerobic or radiobiologically hypoxic cells.

### Spheroids as an *in vitro* model for antibody-targeted therapy of neuroblastoma micrometastases

K.A. Walker, T. Murrey, T.E. Wheldon, A. Gregor & I.M. Hann

*Radiobiology Group, Belvidere Hospital; Radioisotope Dispensary, Western Infirmary and Royal Hospital for Sick Children, Glasgow, UK.*

One of the clinical characteristics of neuroblastoma is its rapid dissemination and the formation of micrometastases. Children with metastatic disease have very poor prognosis and new forms of treatment are being sought. Radioimmunotherapy is a new approach which is under consideration. Laboratory models of radioimmunotherapy of micrometastases may help in the assessment of this strategy.

We have developed an *in vitro* model for micrometastases with a neuroblastoma cell NBI-G which can be initiated to grow as spheroids. Regrowth of these spheroids was assessed following incubation with $^{131}I$ conjugated to a neuroectodermal specific monoclonal antibody UJ13A. The effect of UJ13A-$^{131}I$ on spheroid growth was found to be far greater than that of $^{131}I$ alone, and a dose-response relationship was obtained. In addition penetration of antibody into multicellular spheroids has been investigated.

This model therefore provides a method for studying antibody-targeted therapy and should be useful for the evaluation of alternative antibodies and radionuclides.

**Studies of antibody penetration in cellular spheroids**

J. Carlsson & B. Larsson

*Dept. Phys. Biol., Inst. Rad. Res., Box 535, S-75121 Uppsala, Sweden.*

The penetration of antibodies against CEA, P97 and some other antigens into different types of human tumour spheroids was analysed. All tested antibody preparations penetrated 200–400 $\mu$m into the spheroids within 15 min. No dramatic differences were seen between different types of antibodies or between different types of spheroids when the initial penetration within 15 min was considered. However, after 60 min there was a pronounced accumulation of CEA antibodies in the necrotic area of HT29 spheroids. Such accumulations were not seen in other tested combinations of antibodies and spheroids.

**Multicell tumour spheroid model of radioimmunotherapy**

C.S. Kwok, R. McFadden & S.K. Liao

*Hamilton Regional Cancer Centre, Ontario Cancer Foundation, and McMaster University, Hamilton, Ontario, Canada L8V 1C3.*

*In vitro* multicell tumour spheroids from a human melanoma cell line and a human colon adenocarcinoma cell line (used as control) have been established as a model of poorly vascularized micrometastases *in vivo*. Detailed uptake kinetics by the spheroids of two anti-melanoma monoclonal antibodies (MAb) and a non-specific MAb was investigated. The MAb showed a wide range of reactivity against the melanoma cell but they all had negligible binding with the colon cell. Penetration of the MAb in the spheroids after different incubation times was examined by autoradiography. By using high specific radioactivity $^{131}$I-labelled MAb, therapeutic effects in growth delay and regression on the spheroids were observed. Mathematical modelling of the uptake of the MAb by the spheroids and of the radiation effect on cells of the spheroids is therefore feasible although such micrometastases may not be imaged *in vivo*. Present findings will also provide information to the optimization of other forms of MAb targeted therapy.

**The spheroid as a model for radioimmunotherapy research**

V.K. Langmuir, J.K. McGann & R.M. Sutherland

*University of Rochester Cancer Center, Rochester, NY 14642, USA.*

Multicell spheroids of human colon cancer (HCC) are being used as a model for studies in radioimmunotherapy (RIT). CO112 and WiDr, both well-differentiated HCCs, and 3 different IgG1 monoclonal antibodies (MAbs) to carcinoembryonic antigen (CEA), and their F(ab')2 and Fab fragments are being used. Autoradiography of spheroids after 4 h incubation with 125I-labelled MAb showed heterogeneous binding of MAb. Most of the MAb was on the surface of the spheroid with some MAb penetrating 1–3 cell layers for the intact MAb and up to 6–8 cells for the fragments. Preincubation with cholesterylhemisuccinate or preirradiation with 6 Gy external beam gamma-rays enhanced MAb binding 14–55%. RIT experiments were performed using 131I-labelled MAb at a specific activity of 10–20 mCi mg$^{-1}$ MAb. Maximum binding with MAb 25 was 2.25 ng mm$^{-2}$ spheroid surface area for CO112 and 1.01 for WiDr. Theoretical maximum dose rates achieved, 0.2 mm from the spheroid surface, were 12 cGy h$^{-1}$ for CO112 and 5.4 cGy h$^{-1}$ for WiDr. Even at this low dose rate surviving fraction of WiDr spheroid cells after exposure to labelled MAb 25 was reduced by a factor of about 4 when compared with labelled nonspecific control $IgG_1$ after 72 h (calculated dose = 6 Gy). Cytotoxicity relative to antibody or fragment location, fractionation of antibody doses, and effects when combined with other agents to enhance radiation damage are being evaluated.

The organisers are most grateful to the following commercial organisations who have made contributions towards the costs of the meeting: Analytical Measuring Systems; Becton Dickinson U.K. Limited; Cambridge Medical Books; Flow Laboratories Limited; Gibco Limited; I.C.I. Pharmaceuticals and Techne Cambridge Limited.